# An Azabisphosphonate-Capped Poly(phosphorhydrazone) Dendrimer for the Treatment of Endotoxin-Induced Uveitis

**DOI:** 10.3390/molecules18089305

**Published:** 2013-08-05

**Authors:** Séverine Fruchon, Anne-Marie Caminade, Claire Abadie, Jean-Luc Davignon, Jean-Marc Combette, Cédric-Olivier Turrin, Rémy Poupot

**Affiliations:** 1 INSERM, U1043, CNRS, U5282, Université de Toulouse, UPS, Centre de Physiopathologie de Toulouse-Purpan, Toulouse F-31300, France; E-Mails: severine.fruchon@inserm.fr (S.F.); jean-luc.davignon@inserm.fr (J.-L.D.); 2 CNRS, LCC (Laboratoire de Chimie de Coordination) 205 route de Narbonne, BP 44099, F-31077 Toulouse cedex 4, France; E-Mail: caminade@lcc-toulouse.fr; 3 Solid Drug Development SA, 8 rue John Grasset, Geneva CH-1205, Switzerland; E-Mails: cabadie@soliddrugdevelopment.com (C.A); jmcombette@soliddrugdevelopment.com (J.-M.C)

**Keywords:** PPH [poly(phosphorhydrazone)] dendrimer, anti-inflammatory, uveitis, toxicity

## Abstract

Over the last decade, different types of dendrimers have shown anti-inflammatory properties in their own right. In particular, we have shown that poly(phosphorhydrazone) (PPH) dendrimers are able to foster an efficient anti-inflammatory response in human monocytes and can resolve the main physiopathological features of chronic arthritis in mice at 1 mg/kg. Here we afford new insights into the therapeutic potential of an azabisphosphonate-capped dendrimer (dendrimer **ABP**). We have challenged its anti-inflammatory and immuno-modulatory properties in a robust rat model of acute uveitis induced by lipopolysaccharide (LPS). We show that dendrimer **ABP** at 2 µg/eye is as efficient as the “gold standard” dexamethasone at 20 µg/eye. We have demonstrated that the effect of dendrimer **ABP** is mediated at least through an increase of the production of the anti-inflammatory Interleukin(IL)-10 cytokine.

## 1. Introduction

**Figure 1:**
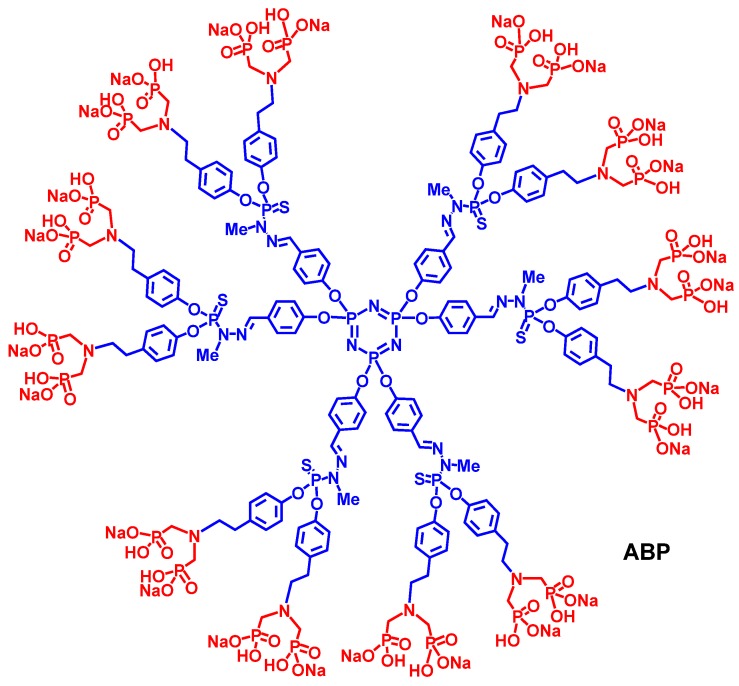
Structure of dendrimer **ABP**. Core (cyclotriphosphazene) and branches (phosphorhydrazones) are in blue, surface end groups (azabisphosphonates) are in red.

Poly(phosphorhydrazone) (PPH) dendrimers are synthesized according to a simple and efficient divergent strategy involving phosphorus-containing building blocks; and their surface functions can be easily modified to afford rationally-designed dendrimers [1]. In this regard, PPH dendrimeric structures have been designed for therapeutic applications like anti-HIV or anti-prion properties, for bio-sensing, drug delivery (transfection agents) or for the bio-imaging of blood vessels to name a few [2]. PPH dendrimers with specific phosphonate surface functions can also favor human immune cell growth. In this respect, we have shown that PPH dendrimers, in particular a first generation dendrimer capped with twelve azabisphosphonate groups, namely dendrimer **ABP** (Figure 1), can activate the *in vitro* proliferation of human Natural Killer (NK) cells (these cells play a key role in fighting against viral infections and cancer) [3]. We have also demonstrated that dendrimer **ABP**, activates human monocytes *in vitro* [4] toward an anti-inflammatory response [5]; and dendrimer **ABP** has emerged as an anti-inflammatory “lead” dendrimer after the screening of the *in vitro* bio-activity of almost eighty dendrimers of different series [6,7,8].

So far, only a few types of dendrimers have anti-inflammatory properties *per se* [9]. In 2011, we demonstrated the *in vivo* anti-inflammatory effects of dendrimer **ABP** in two mouse models of experimental arthritis: the IL1ra-/- and K/BxN models [10]. In IL1ra-/- mice, marked effects are observed on paw swelling, arthritic and histo-pathological scores after intravenous administration of dendrimer **ABP** at 1 and 10 mg/kg weekly (for 12 weeks). Moreover, serum concentrations of pro-inflammatory cytokines and matrix metallo-proteases decreased significantly during treatment. We have also shown that *per os* administration of dendrimer **ABP** at 10 mg/kg/week for twelve weeks resolves experimental arthritis in this mouse model [11]. The prophylactic and therapeutic effects of dendrimer **ABP** have also been demonstrated in the K/BxN serum transfer mouse model [11]. As a result, the lead dendrimer **ABP** has become a serious drug candidate and is currently in pre-clinical development for the treatment of rheumatoid arthritis (RA) and potentially other inflammatory diseases [12,13,14]. In view of consolidating the position of dendrimer **ABP** in this highly competitive market, and to accelerate the bench to market process, it appeared appropriate to evaluate the activity of this compound in a relevant acute disease necessitating topical administration. In this regard, we have chosen the Endotoxin-Induced Uveitis (EIU) in the rat. This model is considered as a clinically relevant model for human anterior uveitis [15,16,17]. It consists in the systemic administration of lipopolysaccharide (LPS) which results in an acute inflammatory response in the anterior and posterior segments of the eye with a breakdown of blood-ocular barrier and inflammatory cell infiltration. Clinical signs of EIU reflect the changes observed in human disease [18]. The work reported here describes the therapeutic effect of dendrimer **ABP** in the robust model of EIU in rats, in comparison with the “gold standard” dexamethasone.

## 2. Results and Discussion

### 2.1. Ocular Tolerability of Dendrimer **ABP**

First, we have assessed the ocular tolerability of dendrimer **ABP** for seven consecutive days following a single intra-vitreal injection in both eyes of rats. Three groups of three male Sprague-Dawley rats have been set up: the first group received the saline vehicle; the second and third groups received 20 µg (low-dose group) and 100 µg (high-dose group) of dendrimer **ABP** per eye, respectively.

During the study, no mortality and no effect on weight gain have been observed. Clinical observations revealed no detectable adverse effect on vision or eyesight in any of the treated animals. Gross clinical signs were limited to ocular observations of cloudiness in the vitreous of all animals of the high-dose group from day 1 to the end of the study, confirmed at necropsy. A slight cloudiness in the vitreous of one low-dose treated animal out of three was also noted from day 2 to the end of the study, but this cloudiness was not confirmed at necropsy two days later. Thus, cloudiness was found to be systematic and more important and persistent in the high-dose group than in the low-dose group.

After dosing, McDonald-Shadduck scores elaborated from funduscopies and slit-lamp examinations (SLE) were consistently zero, with two exceptions when fluorescein staining of the cornea (indicating a disruption of the corneal epithelium) was seen in one eye of an animal of the high-dose group at day 1 and in one eye of the low-dose group at day 6. The lack of any corneal findings during SLE examinations or histological studies suggests that the fluorescein staining did not indicate any underlying pathology but are likely spontaneous incidental background findings and not dendrimer **ABP**-related.

Exhaustive histological studies were performed on eyeball tissue sections (Table 1), and it was found that all animals treated with low dose were within normal limits, whereas examination of tissue sections from the high-dose animals revealed sub-acute to acute inflammation in the vitreous consisting of dense proteinaceous material and inflammatory infiltrate of neutrophils with fewer mononuclear cells.

**Table 1:** Histological inflammatory scores in vitreous (Group 1/Vehicle, Group 2/Low dose: 20 µg/eye, Group 3/High dose: 100 µg/eye. RE: right eye, LE: left eye). Score: 0 = no inflammation, 1 = minimal inflammation, 2 = mild inflammation, 3 = moderate inflammation, 4 = severe inflammation.

Group/Animal number	score		Group/Animal number	score
Group 1/102 (RE)	0		Group 3/301 (RE)	3
Group 1/102 (LE)	0		Group 3/301 (LE)	1
Group 1/103 (RE)	0		Group 3/302 (RE)	2
Group 1/103 (LE)	0		Group 3/302 (LE)	3
Group 2/201 (RE)	0		Group 3/303 (RE)	2
Group 2/201 (LE)	0		Group 3/303 (LE)	2
Group 2/203 (RE)	0		Group 3/304 (RE)	2
Group 2/203 (LE)	0		Group 3/305 (LE)	1

Taking into account these data, we have considered the 20 µg/eye dose as a No Observed Adverse Effect Level (NOAEL) dose of dendrimer **ABP**, while the 100 µg/eye dose was associated with inflammation in the vitreous. Having in hands these results, we have then decided to gain the “Proof of Concept” of the efficacy of dendrimer **ABP** in ophthalmology by the intra-vitreal route in a rat model of Endotoxin-Induced Uveitis (EIU). Increasing doses were chosen according to the tolerability study: 2, 10, 20 and 60 µg/eye, the latter being the only value over the NOAEL.

### 2.2. Efficacy of Dendrimer **ABP** to Treat Endotoxin-Induced Uveitis in Rats

**Figure 2:**
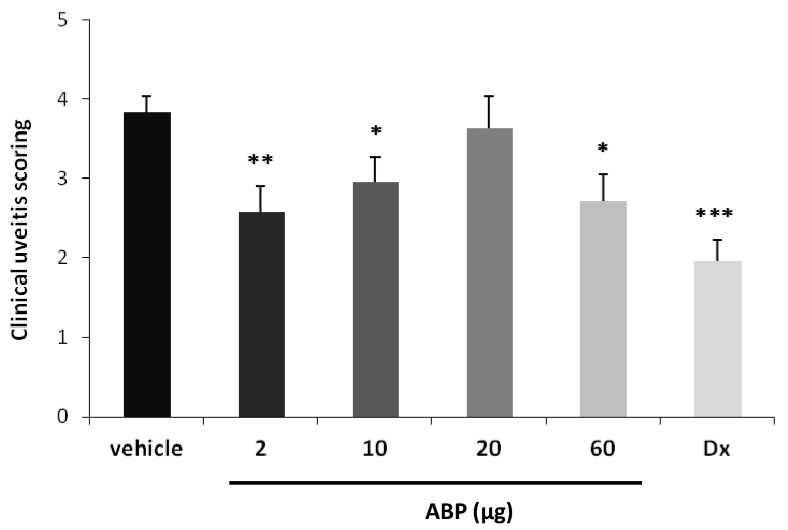
Clinical scoring 24 h after EIU induction and intra-vitreal dendrimer **ABP** administration. Dx is for dexamethasone at 20 µg/eye. Histogram bars represent the mean clinical scoring (arbitrary units) + SEM. * *p* < 0.05, ** *p* < 0.01, *** *p* < 0.001 *vs.* saline vehicle.

EIU is induced with injection of bacterial lipopolysaccharide (LPS) in the foot-pad of female albino Lewis rats and clinical scores are evaluated 24 h after induction. We have evaluated the effect of dendrimer **ABP** in this model of acute inflammatory disease and challenged the “gold standard” dexamethasone. Six groups of six animals (*n* = 12 eyes) have been studied: vehicle only (group 1), dendrimer at 2 µg/eye (group 2), dendrimer at 10 µg/eye (group 3), dendrimer at 20 µg/eye (group 4), dendrimer at 60 µg/eye (group 5), dexamethasone at 20 µg/eye (group 6). Clinical scores of the six groups are presented in Figure 2. One eye in the 10 μg dendrimer **ABP**-treated group has been excluded due to important hyphema, which could potentially modify clinical scoring, which led to work with *n* = 12 in all groups except in group 2 (*n* = 11).

The mean clinical score evaluated 24 h after intra-vitreal saline vehicle administration was 3.83 ± 0.21. The mean clinical score of dendrimer **ABP**-treated eyes was statistically different from the mean score of vehicle group for the doses 2, 10 and 60 μg/eye with 33% (*p* < 0.01), 23% (*p* < 0.05) and 29% (*p* < 0.05) reduction, respectively. The mean clinical score of both eyes from dexamethasone-treated group was statistically reduced compared to the score from saline vehicle treated group with 49% (*p* < 0.001). Thus, these results show that a single intra-vitreal administration of dendrimer **ABP** at the time of LPS injection induces a significant reduction of ocular inflammation at the doses of 2, 10 and 60 µg/eye but does not exert any anti-inflammatory effect at the dose of 20 µg/eye, suggesting an inverse dose-response effect. It is important to note that the tolerability study revealed that administration of the molecule could induce sub-acute inflammation at the site of injection. Considering the small time scale of the assay (24 h), one can assume that the intrinsic anti-inflammatory properties of dendrimer ABP can be temporarily thwarted by the topical administration. Despite the fact that this effect could not be fully rationalized, this series of results show that in the most favorable case the effect of dendrimer **ABP** is not statistically different from the effect of the “gold standard” dexamethasone. In fact, the difference in the mean values of the two groups is not great enough to reject the possibility that the difference is due to random sampling variability (*p* = 0.1455).

**Figure 3:**
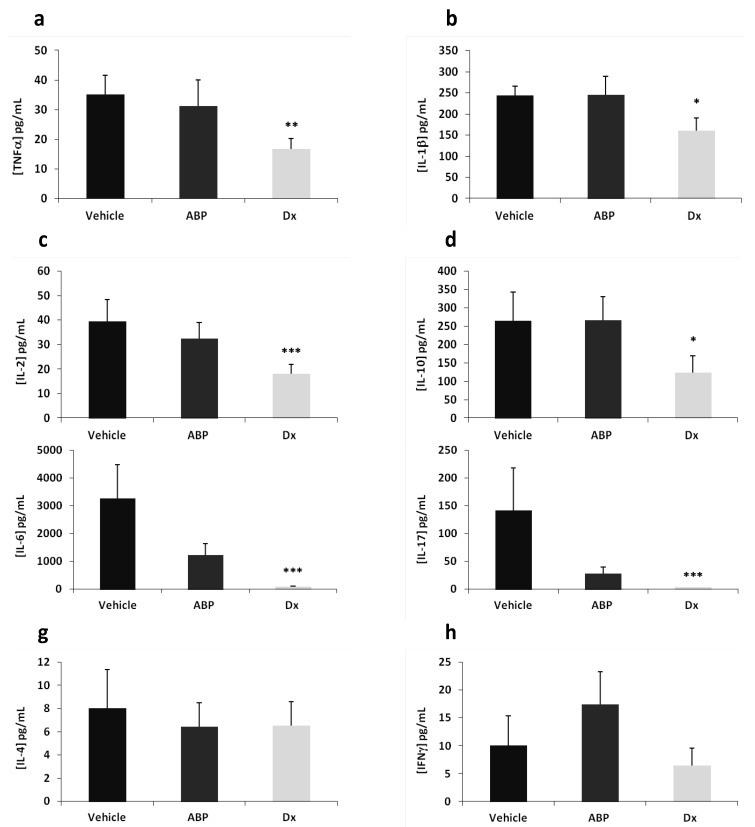
Cytokine concentrations (pg/mL) in aqueous humor and vitreous. Multiplex analysis was achieved 24 h after EIU induction. Dendrimer **ABP** was administered at 2 µg/eye. Dx is for dexamethasone at 20 µg/eye. (**a**) TNFα, (**b**) IL-1β, (**c**) IL-2, (**d**) IL-10, (**e**) IL-6, (**f**) IL-17, (**g**) IL-4 and (**h**) IFNγ. Histograms represent the mean of concentration measurements in each eye + SEM. * *p* < 0.05, ** *p* < 0.01, *** *p* < 0.001 *vs.* saline vehicle.

As the most important and significant effect is obtained with the dose of 2 µg/eye of dendrimer **ABP**, we have assessed its effect at this dose by measuring local (in the aqueous humor and vitreous) and systemic (in the serum) concentrations of pro- and anti-inflammatory cytokines. Cytokine concentrations measured in aqueous humor and vitreous are presented in Figure 3.

At 24 h post-induction and treatment, concentrations of pro-inflammatory/inflammatory cytokines TNF-α, IL-1β, IL-2 and anti-inflammatory cytokine IL-10 in ocular fluids were significantly reduced in the dexamethasone-treated group compared to the saline vehicle treated group (at least *p* < 0.05). On the contrary, TNF-α, IL-1β, IL-2 and IL-10 concentrations were not reduced in animals treated with 2 µg/eye of dendrimer **ABP** as compared to the one treated with saline vehicle alone.

**Figure 4:**
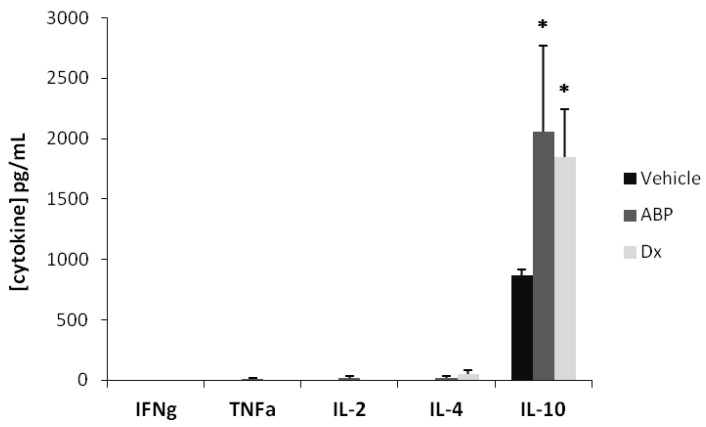
Cytokine concentrations (pg/mL) in serum. CBA analysis was achieved 24 h after EIU induction. Dendrimer **ABP** was administered at 2 µg/eye. Dx is for dexamethasone at 20 µg/eye. Histograms represent the mean of concentration measurements in the serum of each animal + SEM. * *p* < 0.05 *vs.* saline vehicle.

Concentrations of inflammatory cytokines IL-6 and IL-17 in ocular fluids were drastically reduced in the dexamethasone treated group compared to the vehicle treated group (*p* < 0.001), due to the anti-inflammatory effect of the “gold standard” drug. In the dendrimer **ABP**-treated group, IL-6 and IL-17 concentrations tended to be reduced, although this reduction was not found significant. However, the statistical test indicates that the effect of dendrimer-**ABP** at 2 µg is not significantly different from that of dexamethasone (*p* > 0.05). Finally, it was found that the concentration of anti-inflammatory cytokine IL-4 and the concentration of pro-inflammatory cytokine IFN-γ in ocular fluids were not affected by dendrimer **ABP** or dexamethasone treatments at 24 h. In order to evaluate the influence of loco-regional applications on systemic inflammation marker response, we have then assessed the serum concentrations of IFNγ, TNFα, IL-2, IL-4 and IL-10 (Figure 4).

The serum concentrations of pro-inflammatory/inflammatory cytokines IFNγ, TNFα, IL-2 and anti-inflammatory cytokine IL-4 were measured at very low levels when detectable, with no significant difference between the treated and non-treated groups. On the contrary, amounts of anti-inflammatory cytokine IL-10 were detected with a significant increase in the dendrimer **ABP-** and dexamethasone-treated groups. This finding can possibly be related to a trans-location of the drug from the vitreous to the systemic blood pool, which can be related to the small size of dendrimer **ABP**. In this regard, a study on the lymphatic redistribution of dendrimers between blood and lymph suggested that low molecular weight dendrimers can diffuse from capillaries and return to systemic circulation [19].

## 3. Experimental

### 3.1. Synthesis of Dendrimer **ABP**

Dendrimer **ABP** was synthesized as already described [3]. Powder was dissolved in saline solution (NaCl 0.9%). The solution was prepared extemporaneously on the day of administration.

### 3.2. Animals

All animals were treated according to the European Convention and to the Association for Research in Vision and Ophtalmology (ARVO) statements for the use of animals in ophthalmic and vision research [20,21]. Only animals with no visible signs of ocular defects were enrolled. Animals were examined during the pre-test period and particular attention was given to the eyes. They were held in observation for one week before experimentation. Animals were housed individually in standard cages and had free access to food and tap water.

### 3.3. Ocular Tolerability Study

The study consisted of three groups of three male Sprague-Dawley rats. On day 0, animals were weighed, anesthetized and administered by a single 5 µL intra-vitreal injection in both eyes. The first group received the saline vehicle (NaCl 0.9%), the second and third groups received 20 µg (low-dose group) and 100 µg (high-dose group) of dendrimer **ABP** per eye, respectively. On days 0 to 6, at approximately the same time on each day (±1 h), each animal was assessed by clinical observation and eye examinations. Ocular examinations included: funduscopy, slit-lamp examination (SLE) of the cornea using fluorescein dye enabling McDonald-Shadduck scoring. The McDonald-Shadduck Scoring System addresses: conjunctival parameters (congestion, swelling and discharge), aqueous flare (intensity of the Tyndall phenomenon) as presumptive evidence of breakdown of the blood-aqueous barrier; injection of secondary and tertiary vessels in the iris; cloudiness, relative area thereof, neo-vascularization and epithelial integrity (fluorescein labeling) of the cornea; integrity of the lens. On day 8, *i.e.*, 2 days after the final ocular examination, all of the animals were sacrificed. Eyes were collected at necropsy, fixed in modified Davidson’s solution for 12 h, followed by 10% neutral buffered formalin and processed for histology. Hematoxylin-Eosin stained tissue sections were evaluated via light microscopy by a board-certified veterinary pathologist.

### 3.4. “Proof of Concept” in an EIU Rat Model

Thirty-six female albino Lewis rats were randomly divided into six groups of six animals each. EIU was induced by a 100 µL footpad injection of sterile pyrogen-free saline solution containing 200 µg of LPS (lipopolysaccharide from *Salmonella typhimurium*, Sigma-Aldrich, Saint-Quentin, France). Animals were treated immediately before EIU induction by a 5 µL intra-vitreal injection in both eyes of a saline solution (NaCl 0.9%) containing no active ingredient (group 1), or 2 µg of dendrimer **ABP** (group 2), or 10 µg of dendrimer **ABP** (group 3), or 20 µg of dendrimer **ABP** (group 4), or 60 µg of dendrimer **ABP** (group 5), or 20 µg of dexamethasone (group 6). Animals were examined by slit-lamp (SLE) at 24 h, *i.e.*, the clinical peak of the disease in this model. The intensity of clinical ocular inflammation was scored on a scale from 0 to 5 for each eye. Grade 0 indicates no inflammation. Grade 1 indicates the presence of a minimal iris and conjunctival vasodilatation but without the observation of flare or cells in the anterior chamber (AC). Grade 2 indicates the presence of moderate iris and conjunctival vessel dilation but without evident flare or cells in the AC. Grade 3 indicates the presence of intense iris vessel dilation, flare and less than ten cells per slit-lamp field in the AC. Grade 4 indicates the presence of more severe clinical signs than Grade 3, with more than ten cells per slit-lamp field in the AC, with or without the formation of a hypopyon. Grade 5 indicates the presence of intense inflammatory reaction, fibrin formation in the AC and total seclusion of the pupil. Clinical evaluation was performed in a blinded manner. At the end of experiment, *i.e.*, 24 h after LPS challenge, rats were anesthetized by intra-peritoneal injection of pentobarbital (30 mg/kg, Sanofi-Aventis, Paris, France) then killed with a lethal dose of pentobarbital.

### 3.5. Measurement of Cytokine Concentrations in Ocular Fluids

Aqueous humor and vitreous from both eyes of each animal were taken after sacrifice. Pro-inflammatory T helper cytokines TNFα, IL-1β, IL-2, IL-6, IL-17 and IFNγ as well as anti-inflammatory cytokines IL-4 and IL-10 quantities were determined by Multiplex analysis (Milliplex Map Kit; Millipore, Saint-Quentin-en-Yvelines, France).

### 3.6. Measurement of Cytokine Concentrations in Serum

Sera from the three groups of rats (saline vehicle, **ABP** 2 µg and dexamethasone) were collected at the end of the experiment and stored at −80 °C. They were used for the simultaneous determination of five cytokine (IFNγ, TNFα, IL-2, IL-4 and IL-10) levels with Cytometric Bead Array (rat CBA Flex set, BD Biosciences, San Jose, CA, USA) on a FACS Calibur flow cytometer (BD Biosciences) according to the manufacturer’s instructions. The amounts of each of the cytokines were analyzed in relation to standard curves using the FCAP Array software (BD Biosciences).

### 3.7. Statistical Analyses

Results were expressed as mean + SEM (Standard Error of the Mean). Data were compared with adequate non parametric Kruskal-Wallis ANOVA on Ranks to assess statistical significance with Sigma Stat software (Systat Software, San Jose, CA, USA). *p* < 0.05 was considered statistically significant. * *p* < 0.05, ** *p* < 0.01, *** *p* < 0.001.

## 4. Conclusions

Previously we have shown that dendrimer **ABP** has anti-inflammatory and immuno-regulatory therapeutic effects in a mouse model of chronic arthritic inflammation when administered by systemic routes [11], making it a drug-candidate for the treatment of chronic inflammatory diseases such as rheumatoid arthritis [10]. In this work we have investigated the anti-inflammatory response of dendrimer **ABP** in a robust model of acute inflammation, namely the EIU in rats, performing a loco-regional administration of the molecule by the intra-vitreal route.

The tolerability of the dendrimer **ABP** was evaluated after ocular administration in rats. The NOAEL dose was established at 20 µg/eye. The intensity of the clinical ocular EIU disease was evaluated by funduscopy and SLE 24 h after induction by LPS injection. We have shown that a single intra-vitreal administration of dendrimer **ABP** at the time of LPS injection induces a significant reduction of ocular inflammation. The strongest effect was observed at the lowest dose (2 µg/eye), and the effect dendrimer **ABP** was not found to be statistically different from the effect of the “gold standard” dexamethasone at higher dose (20 µg/eye).

We have also demonstrated that cytokine dosage in ocular fluids is also consistent with an anti-inflammatory effect of dendrimer **ABP** at 2 µg/eye. IL-6 and IL-17 concentrations tend clearly to be reduced by this low dose, even though the reduction observed is not significant. Nevertheless, dendrimer **ABP** does not down-regulate important pro-inflammatory cytokines such as TNFα and IL-1β. Moreover, in serum, both dexamethasone and dendrimer **ABP** induced a strong increase of IL-10, the paradigm of anti-inflammatory and immuno-modulatory cytokines. This latter result suggests that these anti-inflammatory drugs might undergo a systemic passage while administered by a loco-regional route [22,23].

In the EIU rat model, the LPS administration may induce ocular inflammation by stimulating the production of pro-inflammatory cytokines by activated monocytes/macrophages [17]. We have already shown that dendrimer **ABP** targets monocytes and triggers their anti-inflammatory activation [5,7]. Therefore, the anti-inflammatory effect of dendrimer **ABP** in the EIU model may be mediated through anti-inflammatory activation of monocytes/macrophages, leading to systemic IL-10 production. Moreover, we have also shown that dendrimer **ABP** inhibits human CD4+ T lymphocytes [24], these cells can act as inflammatory players in the physiopathology of EIU in rats and may also be targeted and regulated by dendrimer **ABP** in this model.

Taken together these results indicate that topical administration of dendrimer **ABP** has a significant anti-inflammatory activity in a clinically relevant rat model of anterior uveitis. This idea is supported by the fact that dendrimer ABP at 2 µg/eye is as active as “gold standard” dexamethasone at 20 µg/eye. This strengthens the anti-inflammatory and immuno-regulatory properties of dendrimer **ABP** for both chronic and acute inflammatory diseases, and makes it a possible drug-candidate for inflammatory diseases in ophthalmology.
